# Novel Approach in the Use of Plasma Spray: Preparation of Bulk Titanium for Bone Augmentations

**DOI:** 10.3390/ma10090987

**Published:** 2017-08-24

**Authors:** Michaela Fousova, Dalibor Vojtech, Eva Jablonska, Jaroslav Fojt, Jan Lipov

**Affiliations:** 1 Department of Metals and Corrosion Engineering, University of Chemistry and Technology Prague, Technicka 5, 166 28 Prague 6, Czech Republic; dalibor.vojtech@vscht.cz (D.V.); jaroslav.fojt@vscht.cz (J.F.); 2 Department of Biochemistry and Microbiology, University of Chemistry and Technology Prague, Technicka 5, 166 28 Prague 6, Czech Republic; eva.jablonska@vscht.cz (E.J.); jan.lipov@vscht.cz (J.L.)

**Keywords:** titanium, plasma spray, porosity, bone augmentation

## Abstract

Thermal plasma spray is a common, well-established technology used in various application fields. Nevertheless, in our work, this technology was employed in a completely new way; for the preparation of bulk titanium. The aim was to produce titanium with properties similar to human bone to be used for bone augmentations. Titanium rods sprayed on a thin substrate wire exerted a porosity of about 15%, which yielded a significant decrease of Young′s modulus to the bone range and provided rugged topography for enhanced biological fixation. For the first verification of the suitability of the selected approach, tests of the mechanical properties in terms of compression, bending, and impact were carried out, the surface was characterized, and its compatibility with bone cells was studied. While preserving a high enough compressive strength of 628 MPa, the elastic modulus reached 11.6 GPa, thus preventing a stress-shielding effect, a generally known problem of implantable metals. U-2 OS and Saos-2 cells derived from bone osteosarcoma grown on the plasma-sprayed surface showed good viability.

## 1. Introduction

Orthopaedic surgery requires the use of bone grafts to help musculoskeletal injuries to heal. Although autologous grafts are the best solution, problems related to bone harvesting force surgeons to seek bone graft substitutes. To be a substitute for bone, a material is supposed to mimic its natural properties. From a wide range of materials possible for medical use inside the living body, metals are the most suitable for load-bearing applications in orthopaedics [1]. Despite their great strength, bulk metals are still not ideal. Compact structures do not allow the bone tissue to grow in so the implant fixation is limited during the healing process. Also, over time, thinning of the surrounding bone tissue may be induced by the higher stiffness of the metals taking over the physiological load [2,3]. Therefore, porous structures are desirable. There are different methods of preparing porous metals, some of which are already very sophisticated, providing control over pore size, shape, distribution, orientation, or building gradient in porosity [2,4,5,6]. However, for the preparation of simple bone augmentations for filling bone defects, the fastest, cheapest, and simplest way is desirable.

In our paper, we decided to employ plasma spray technology to prepare augmentations or scaffolds for biomedical use. When there is a lack of bone tissue as a result of some pathological state or serious injury, the resulting void needs to be filled [7]. A total hip endoprosthesis in an elderly patient could serve as an example. If the joint acetabulum is damaged, the acetabular cup cannot be fixed in its position securely. The bone defect, therefore, needs to be filled by a proper augmentation. Such an augmentation must mimic the behavior of original bone in terms of mechanical performance. Particularly, a mismatch in elasticity is problematic. Much higher stiffness (represented by elastic modulus) of the implanted material compared to the treated bone may lead to an aseptic implant loosening because the surrounding bone tissue is not loaded sufficiently and resorbs consequentially [8].

To prevent stress-shielding, porous materials are often used. Although the primary function of porosity in orthopaedic metallic implants is to support tissue adhesion, growth, and vascularization, another desirable property is the ability to control their elastic modulus to match that of bone. Not only limited stress-shielding, but also improved biological fixation thanks to the bone tissue ingrowth through the pores, promote implant lifespan in vivo when porous materials are employed [8]. There have been many studies showing that appropriate surface design can improve early implant stability and induce an accelerated healing response [9,10].

Due to the fact that plasma-sprayed titanium coatings have been already used to promote the osseointegration of bone implants [11,12,13,14,15], we decided to attempt to take advantage of reducing the elastic modulus too and, therefore, prepared bulk material by thermal plasma-spray technology. There are not many similar studies in the literature. Some papers deal with applying a spray technique for the preparation of thick titanium layers or bulks but the majority of papers concern a cold spray process in which particles of high kinetic energy are plastically deformed via an adiabatic shearing process and adhere to the substrate surface without the use of heat. The advantages of cold spray over thermal spray are eliminated issues of oxidation, distortion, and phase transformation [16,17,18]. We employed thermal spray because that is an accessible technology, known for more than 100 years, while cold spray has begun to be intensively studied and improved only in recent years. Moreover, because of high gas flows, high pressures (up to 3.5 MPa) being necessary for particles to reach supersonic velocities for the successful deposition or use of helium; thermal spray is favored by a significantly lower cost [19]. The advantage of traditional thermal spray processes also lies in an extremely wide range of deposit materials, as well as substrate materials. Even non-melting materials (e.g., graphite, carbides) can be co-deposited to create unique composite materials with mixed, layered, or gradient microstructures. Such composites can then bring great functionality to new products. For example, the addition of hydroxyapatite may be beneficial for bone reparative materials. Possible substrates include even polymers, wood, or paper. Conversely, the cold spray is essentially limited to depositing ductile materials onto hard substrates. Composites can be prepared uneasily and with poor adhesion. Finally, when considering the processing of extremely reactive titanium, it may still react with oxygen, although it is true for most materials that cold spray deposits boast no or minimal oxidation. Therefore, cold spray is questionable here [20].

In Ajaja et al. [16], variability in the spraying porosity due to the dependence on the velocity of powder deposition was discussed. The highest porosity of a thick titanium coating (17 ± 5%) was reached at the lowest tested velocity of 648 m/s. For the highest deposition velocity, cold spray was shown to produce coatings behaving in a similar manner to a bulk material but with higher hardness within particles due to residual stresses brought about by the deposition impact. Sun et al. [21] prepared Ti porous coating using a Mg holder. With 48.6% porosity, cortical bone behavior was approached. Jaeggi et al. [22] employed vacuum plasma spray to prepare open porous Ti coatings of a thickness up to several millimeters for biomedical implant application. The work focused on pre-alloying the titanium powder with Si to increase the mechanical strength of coatings with porosity up to 60% and showed that the shear strength could be increased by a factor of about two by the use of the surface-alloyed precursor powder.

This paper brings a very comprehensive study of the first set of specimens, starting with a simple structural characterization, then a mechanical properties assessment, and finishing with in vitro tests of compatibility with human cells derived from osteosarcoma. Based on the results, it shows that the novel approach of using plasma spray for the preparation of bulk titanium is effective and can be considered for applications in bone surgery.

## 2. Results

### 2.1. Surface

**Figure 1:**
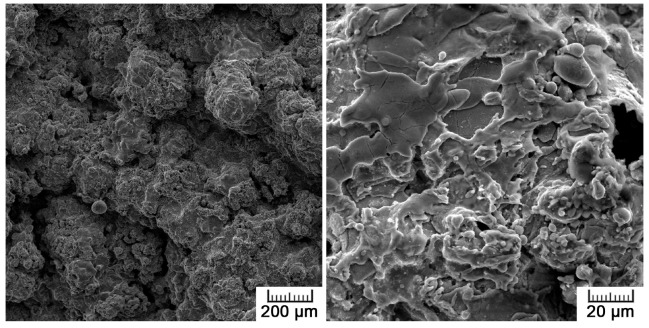
Surface of plasma-sprayed titanium.

Figure 1 shows the surface morphology of the prepared sample, characteristic of plasma spraying. The surface is very rough, formed by overlapping droplets of solidified deposited titanium. Immediately after the impact of the molten powder particles against the substrate, the droplet is flattened and spread out in the radial direction. A thin layer of the drop, which is in contact with the substrate, is solidified during spreading. The residual liquid jets out and detaches to form satellite droplets, resulting in a so-called ‘splashing’ phenomenon. These small droplets solidify during splashing and land on the substrate or a prior deposit, resulting in the formation of weakly adhered solidified fine droplets around the bulk of the splats [23]. The splashed particles surrounding the spread droplets can be also observed in Figure 1.

The high surface roughness was quantitatively assessed by Ra and Rz parameters. The average roughness of plasma spraying Ra was 26.4 ± 4.0 µm, but the maximum height roughness Rz reached 145.3 ± 22.9 µm, evidencing sharp level jumps alternating with smoother surface sections.

**Figure 2:**
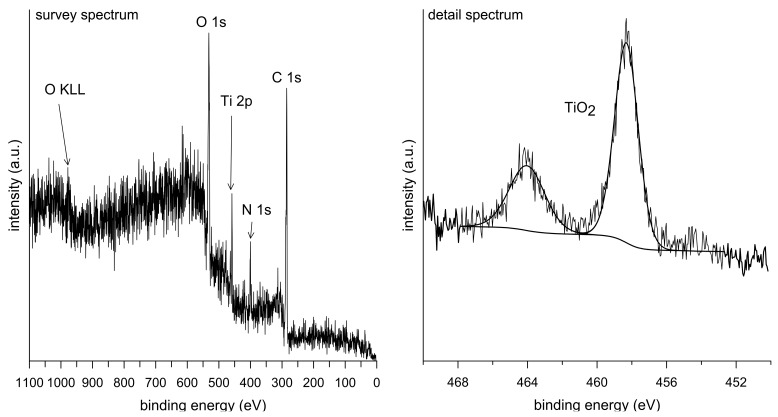
Survey and detail spectra of plasma-sprayed Ti.

The chemical composition of the material surface was determined by XPS (X-ray photoelectron spectroscopy) analysis. The obtained survey spectrum along with a detailed spectrum showing the double peak of titanium oxide are shown in Figure 2. The detected elements in the survey spectrum are Ti, O, C, and N. In the detailed spectrum, it is evidenced that the surface layer consists only of TiO_2_. Carbon and nitrogen are contamination elements from the environment. Due to the high affinity of titanium to oxygen, a thin layer of TiO_2_ (5–10 nm) is formed immediately on its surface when exposed to the air and gives it a passive character [24]. At a high process temperature during the plasma spray, oxidation was further promoted because there was still a certain amount of residual oxygen, although the process was carried out in an argon atmosphere. Therefore, the titanium powder particles were oxidized prior to the deposition.

### 2.2. Structure and Porosity

**Figure 3:**
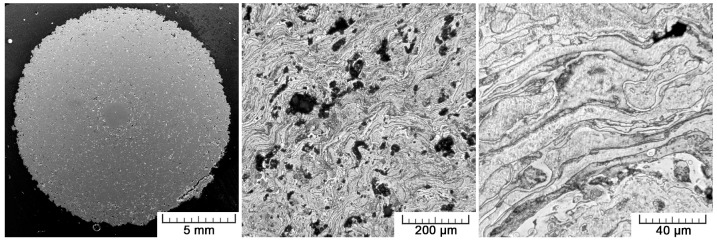
The structure of plasma-sprayed Ti from the macro- to the micro-scale.

**Figure 4:**
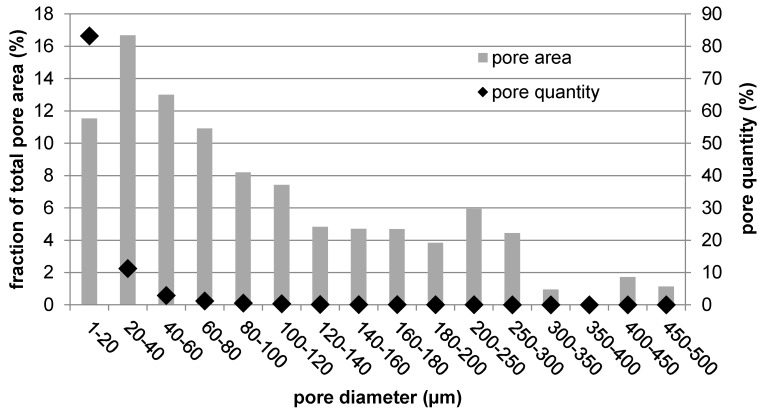
Pore distribution.

Figure 3 shows a cross-section of the prepared sample, along with images of higher magnification. In the middle, the wire that served as a substrate for the plasma spray can be distinguished. It represents 1% of the sample cross-section. The entire plasma-sprayed area surrounding the wire is porous. The pores are evenly distributed over the entire cross-section. The porosity histogram given in Figure 4 shows that the pore size ranges from 1 µm up to 0.5 mm in diameter. The most numerous are the smallest pores of 1 to 20 µm. The total porosity was determined to be 15.4 ± 0.8%, with half of the total pore area being represented by small pores (100 µm at maximum) and half by large pores (100 to 500 µm).

**Figure 5:**
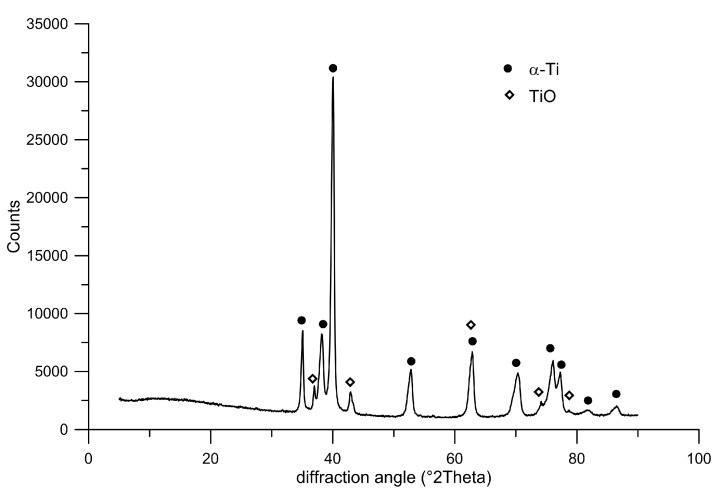
Diffraction pattern showing phase composition of the Ti prepared by plasma spray.

Plasma-sprayed deposits are generally composed of cohesively bonded splats created by the high-rate impact and rapid solidification of a high flux (millions of particles/cm^2^/s) of plasma-melted particles, which result in ‘brick wall’-type microstructures entwined in complex arrays. Such a microstructure can be observed at the highest magnification in Figure 3. Dark interfaces between individual splats are clearly visible. Here, titanium oxides are present, as the surface of the deposited particles was oxidized. The splats themselves are composed only of single α-Ti phase, as pure titanium was used. The phase composition is confirmed by the diffraction pattern shown in Figure 5. The physical properties and behavior of such a deposit are expected to depend on the cohesive strength among the splats, the size and morphology of the porosity, the occurrence of cracks and defects, and, finally, on the microstructures within the splats themselves [25].

### 2.3. Mechanical Properties

**Figure 6:**
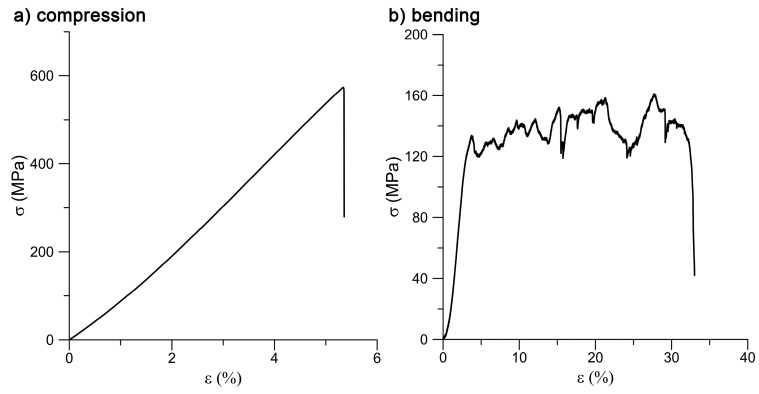
Compressive and bending stress-strain curves.

Figure 6 shows the stress-strain curves for the compressive and bending tests. Regarding the porous nature of the plasma-sprayed material, specimens behave as fragile (similarly to bone), which is declared by the entirely linear character of the compressive stress-strain curve. The obtained mechanical properties are summarized in Table 1.

After the bending, the stress-strain curve is not smooth but is disturbed by stress fluctuations because the material performance was strongly influenced by its porous structure. The testing body gradually disrupted the pore interconnections. Drops in stress were recorded because of the poers being penetrated and the titanium spraying separating from the substrate wire. The wire was bended up to a load of 161 MPa, and then the entire specimen failed. The bending strength of the whole structure is thus 161 MPa.

**Figure 7:**
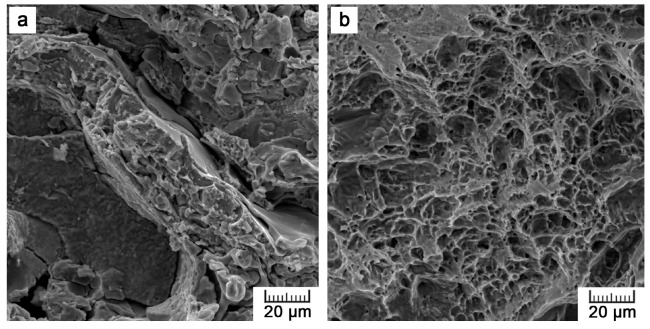
Fractured sample after the bending test: (**a**) plasma-sprayed Ti and (**b**) Ti wire.

Figure 7 shows the fracture surfaces after the mechanical tests. Typical structure of plasma spraying can be observed as it is formed by solidified splattered droplets of sprayed metal that overlap and interconnect into the form of the final bulk material. The fracture surface in the plasma-sprayed part generally exerts a fragile nature but with some signs of plasticity within the splats. A lot of unfractured areas can be distinguished too, as these are parts that were occupied by pores. By contrast, the fracture surface of the internal wire (Figure 7b) is formed by shallow dimples, indicating ductile fracture.

To assess the mechanical performance under dynamic loading conditions, a simple Charpy test was carried out. The measured fracture toughness was 72 kJ/m^2^. Compared to wrought titanium, with a toughness value of 300–660 kJ/m^2^ (according to ASM), the fracture toughness of the plasma-sprayed bulk titanium was significantly decreased. The cause is the porous nature of plasma spraying. Numerous pores not only reduce the load-bearing area but act as stress concentrators especially, which significantly facilitates the crack propagation.

### 2.4. Cytocompatibility

**Figure 8:**
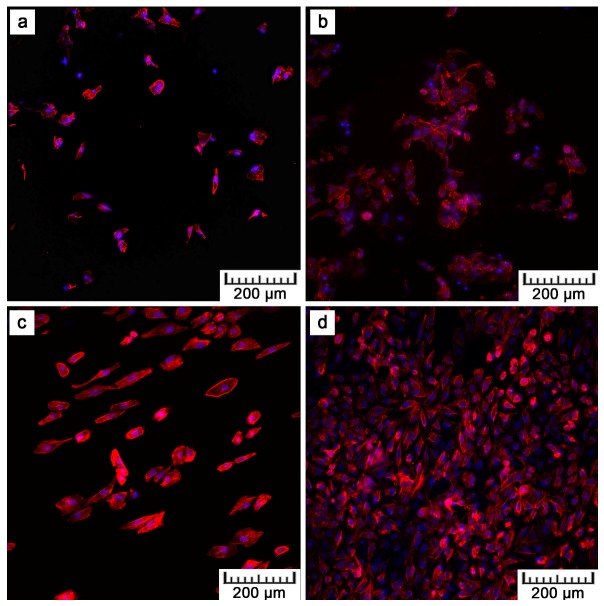
U-2 OS cells cultivated on: (**a**,**b**) the plasma-sprayed titanium; (**c**,**d**) ground Ti6Al4V for one (left images) and four (right images) days (red—F-actin, blue—nuclei, magnification 100×).

**Figure 9:**
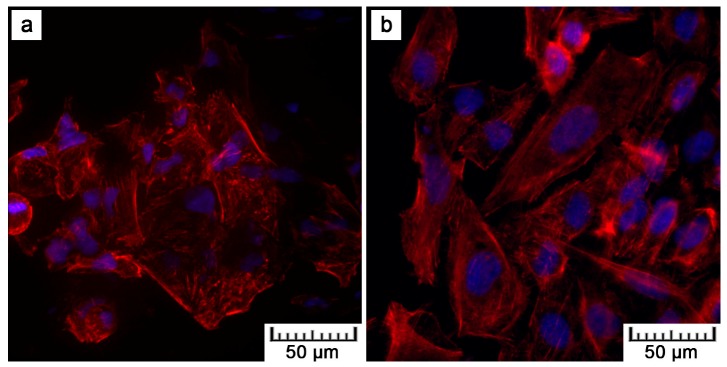
Cell morphology of U-2 OS cells cultivated on: (**a**) the plasma-treated sample; (**b**) ground control for four days (red—F-actin, blue—nuclei, magnification 400×).

**Figure 10:**
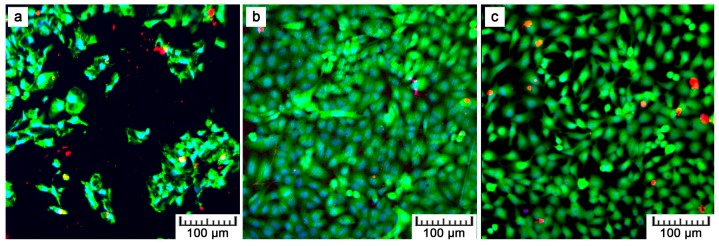
U-2 OS cells cultivated on the (**a**) plasma-sprayed titanium; (**b**) ground control; (**c**) cell culture dish for four days, stained with Calcein-AM and ethidium-homodimer (green—living cells, red—dead cells, blue—nuclei of all cells, magnification 100×).

Figure 8 shows the fluorescence images after the DAPI (4′,6-diamidino-2-phenylindole)/phalloidin staining of the U-2 OS cells after one and four days of cultivation. After four days, the cells proliferated on all materials. Nevertheless, F-actin visualization shows that the cell density on the plasma-treated samples was lower than that on the ground control, which was caused by the coarse surface (Figure 1). However, more detailed images given in Figure 9 evidence the morphology of the cells on the plasma-treated samples as comparable to that of the control. The polyhedral shape, the spreading into a monolayer, and the visible actin network signify vital cell growth (Figure 9a). Moreover, a Live/dead assay with the U-2 OS cells grown on the samples for show days showed a similar phenomenon as F-actin visualization. There was lower amount of cells on the plasma-treated samples in comparison with the controls, but all the samples exhibited good cell viability (Figure 10, live cells stained as green).

## 3. Discussion

Plasma spray processing is a high-velocity impact deposition method that combines the steps of melting, rapid solidification, and consolidation into a single step. It is a well-established method for forming particularly protective coatings but also free-standing bulk near-net shapes. Moreover, its versatility enables the processing of a wide range of metals, alloys, intermetallics, ceramics, and composites [25,26].

In general, the plasma spray coating methods are divided into the vacuum plasma spray (VPS) process and the atmospheric plasma spray (APS) process by the operating pressure ranges. Atmospheric plasma spraying (APS) deposition brings defects such as porosity, oxide inclusions, residual stresses, or unmelted particles. With vacuum plasma spraying (VPS), it is already possible to produce well-adhered dense metallic alloy deposits with minimal oxidation. That is useful, especially for the production of wear- and corrosion-resistant coatings. Nevertheless, the APS drawbacks can be turned into benefits when porous structures are desired. Oxidation during APS increases splat viscosity, and the presence of oxides may increase the splat/substrate interfacial friction. An arrested flow then results in the imperfect bonding of the solidifying droplets, and pores are formed. Therefore, APS was employed in this study [25,27].

When trying to get the metallic material closer to the behavior of human bone, porosity is the key factor. First, it helps to minimize the mismatch in elasticity that is critical for proper bone loading. Second, it is necessary for material integration into the living tissue. There were many studies attempting to determine the ideal pore size in terms of bone cell ingrowth, migration, and nutrients transport. The minimal size was determined as 100 to 150 µm [28], but some works observed promoted bone formation with sizes over 300 µm [29]. Therefore, in general, pores in the range between 100 and 400 µm are suitable for optimal bone growth [30]. About half of the pore area in the prepared titanium rod fell within this range, promoting its biological performance. The second half was represented by the most numerous pores, which were smaller than 100 µm. Although these were not suitable for tissue ingrowth, they contributed to the reduction in mechanical properties down to bone-like properties by lowering the effective load-bearing area, acting as stress concentrators, and promoting crack propagation.

The prepared specimens showed the best mechanical performance under compressive loading. The ultimate compressive strength (UCS) reached 628 MPa, which is a value fairly sufficient for the intended bone applications. Naturally, bone exerts a UCS of about 190 to 245 MPa [31]. On the other hand, while preserving very good strength, the selected technological approach yielded a significant decrease in Young´s modulus. Compared to the 110 GPa usually obtained with bulk titanium, the modulus of elasticity was reduced 10 times. Such an elasticity value already perfectly falls into the range of human bone elasticity, being 7 to 25 GPa [31] depending on the particular bone type. Also in bending, the obtained value of bending strength (BS) fell into the range of human bone. Ascenzi et al. [32] studied behavior of single Haversian systems forming bone and determined the bending strength of this single unit to be 390 MPa. However, as a whole, bone can reach a BS of about 50 to 300 MPa [33], depending on the calcium content. Concerning the resistance to dynamic loading, the reduction in fracture toughness was registered compared to that of conventionally prepared titanium, but still the bone behavior during an impact (2–5 kJ/m^2^ [34]) was exceeded.

Since the studied material is considered for application inside the living body, its biocompatibility is also crucial. As TiO_2_ is formed spontaneously on titanium surfaces and acts as a protective passive layer, giving the material bioinert character, it was possible to assume that the material itself would be suitable for smooth cell growth [35]. Concerning results obtained by XPS analysis, no harmful effect was expected in terms of surface chemistry, which was not negatively influenced by the applied processing route. Another surface property influencing cell adhesion is roughness, which was of concern in the case of the plasma-spray approach. The tested samples exhibited macroroughness as well as microroughness. Macroroughness is defined in the range of 100 μm–milimeters and microroughness in the range of 100 nm to 100 μm. Although the splat surface was relatively smooth, the overall specimen surface was very irregular, which is evidenced by the maximum height roughness of 145.3 ± 22.9 µm. The macroroughness usually has a beneficial effect. On the other hand, the influence of microroughness is controversial; in some cases it promotes cell growth, and, in others, it has a negative impact on cells [36].

By preliminary tests, the in vitro cytocompatibility was assessed. Figure 8, Figure 9 and Figure 10 show that U-2 OS cells adhered and proliferated on the plasma-sprayed titanium. Nevertheless, their number was lower compared to the positive control. That is also confirmed by nuclei counting, the results of which are stated in Table 2. The present values are rather comparative because it was not possible to count all the cells growing on the plasma-spraying surface as a result of its high roughness. Counting was possible only after 24 h of cultivation. After four days, counting was completely disabled because of overlapping nuclei in the z-stack of images. Although we were not able to obtain quantitative information by fluorescence visualization, we obtained a positive finding indicating further cell ingrowth. Some cells were hidden inside the narrow pores opened towards the surface.

**Figure 11:**
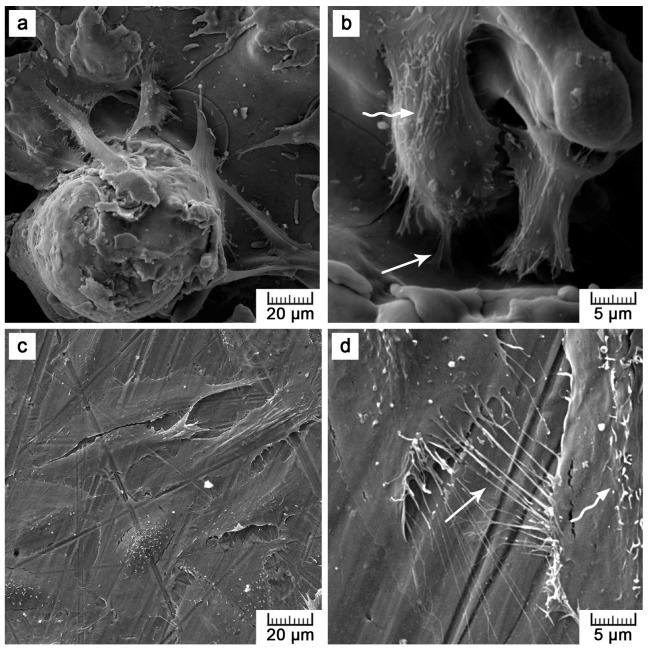
Images of Saos-2 cells cultivated on: (**a**,**b**) the plasma-sprayed titanium and (**c**,**d**) ground control for four days obtained by scanning electron microscopy (arrow—filopodia, wavy arrow—microvilli).

Although lower cell density was observed, the cells exerted healthy morphology (Figure 9). From Live/Dead Assay images, it can be assumed that the 70% viability limit for material cytocompatibility was exceeded as the majority of cells were alive (cells stained in green in Figure 10). To observe cells interacting with the surface and to explain the lower and uneven cell density (Figure 8b), Saos-2 cells seeded onto the flat samples were subjected to scanning electron microscopy (SEM) (Figure 11). SEM revealed that the reason may be the presence of microscale irregularities on the surface. The cells had to bridge these protrusions, which can reduce cell adhesion and spreading. Nevertheless, these cells growing over irregularities were spread and had well-developed filopodia and microvilli (Figure 11b).

Our observations in vitro are in accordance with Martin et al. [37] and Borsari et al. [38]. Martin et al. [37] compared various treatments of Ti, including plasma spray, and stated that the proliferation rate of osteoblast-like MG-63 cells decreased as the surface roughness increased. Borsari et al. [38] prepared Ti coating of different roughness by vacuum plasma spray and observed that the microroughness (Ra = 40 µm) led to reduced contact of the MG-63 cells with the surface. On the other hand, experiments in vivo showed that bone-implant contact and bone–implant strength was positively correlated with the increasing roughness of the implant surface [10,39]. Therefore, it is difficult to predict whether the tested topology would be suitable for this particular application in vivo. However, all over-discussed properties suggest that the plasma-spray approach was selected reasonably and provided the desired results.

## 4. Materials and Methods

### 4.1. Sample Preparation

The titanium samples were prepared by thermal plasma spray using a MP-100-APS plasma spray system (Flame Spray Technologies B.V., Duiven, The Netherlands). The plasma was generated at a voltage of 55 V and a current of 450 A. Argon and hydrogen process gases were supplied at a controlled rate of 60 L/min for Ar and 30 to 40 L/min for H_2_. Titanium powder (Figure S1) with a particle size in the range of 150 to 250 µm was continuously sprayed on a rotating Ti wire (diameter of 1.6 mm, rotation speed of 30 to 60 min^−1^) from a distance of 170 to 190 mm. It concerned titanium sponge metal of 99.5% purity (FLOMASTER™ metal powder, F. J. Brodmann & Co., L. L. C., Harvey, LA, USA). The powder feed rate was 100 g/min. The Ti wire was intensely cooled down by air, and the spraying was kept at the temperature of 343.15 K.

A set of rods of 16.4 ± 0.3 mm in diameter were produced. For biological tests, flat samples were also prepared by coating the Ti6Al4V plates (circle of 45 mm in diameter and 3.7 mm in height) with 1 mm thick layer of titanium (Figure S2).

### 4.2. Sample Characterization

The prepared samples were characterized in terms of their surface morphology and chemical composition, structure, and porosity.

The surface morphology was studied by scanning electron microscopy (SEM, TESCAN VEGA-3 LMU equipped with Oxford instruments INCA 350 EDS analyzer). The surface roughness was determined by Mitutoyo SurfTest SJ400 (JIS B 0601-2001 standard). The Ra (average roughness—the arithmetic average of the absolute values of the roughness profile ordinates) and Rz (mean roughness depth) parameters were statistically evaluated from 10 measurements with a 2.5 mm cut-off length. To determine the chemical composition of the plasma-influenced surface, X-ray photoelectron spectroscopy (XPS) was carried out using an ESCAprobe P (Omicron Nanotechnology Ltd., Hesse, Germany) spectrometer equipped with an Al Kα (λ = 1486.7 eV) X-ray source. An energy step of 0.05 eV was used. The measured spectra were normalized to the binding energy of adventitious carbon (the C1s peak, 285.0 eV). The data for the chemical state evaluation were obtained from the NIST (the National Institute of Standards and Technology) X-ray Photoelectron Spectroscopy Database.

For structure observation, a transverse metallographic section was prepared in a conventional metallographic way using P180-P4000 SiC grinding papers, diamond polishing paste D2, SiO_2_ polishing suspension, and Kroll’s etching reagent. The structure was observed with a light metallographic microscope (Olympus PME3) and SEM. The porosity was assessed by an image analysis in ImageJ software, as well by a gravimetrical approach with the use of the following equation:

(1) $$p=\left(1-\frac{m}{\rho_{\mathrm{th}}\cdot V}\right)\times 100\%$$

where *m* is the weight of the specimen (in g), *ρ*_th_ is the theoretical mass density of titanium (4.5 g·cm^−3^), and *V* is the apparent volume calculated from the external dimension. The phase composition was studied by X-ray diffraction using a PANalytical X´Pert PRO X-ray diffractometer equipped with a Cu anode (XRD).

### 4.3. Mechanical Properties Testing

The mechanical properties of the prepared samples were tested under both static (in compression and bending; CSN EN ISO 7438) and dynamic conditions (impact; CSN EN ISO 148-1). Compressive and bending tests were selected to assess the mechanical behavior because these loading conditions mimic the real loading of augmentations in the bone.

For static tests, a LabTest 5.250SP1-VM universal loading machine was used. The tests were performed at room temperature with a strain rate of 0.001 s^−1^. A compression test was carried out on a cylinder of 14 mm in diameter and 21 mm in height (1.5 ratio). The fracture toughness was determined by a Charpy impact test. The fracture surfaces were subjected to fractographical evaluation on SEM.

### 4.4. Cytocompatibility Assessment

With respect to the intended application in the area of bone reparation materials, the cytocompatibility of the prepared material had to be verified. Flat plates of Ti6Al4V with a 1 mm thick layer of plasma-sprayed titanium were fabricated for this purpose. As a positive control, the same Ti6Al4V plates (treated with P400-1200 SiC grinding paper), but without the coating, were used.

Samples were divided into three groups; for fluorescence microscopy (after one and four days of cultivation, F-actin visualization), for commercial LIVE/DEAD^®^ Viability/Cytotoxicity assay (after four days); and for SEM observation (after four days). Prior to the direct contact tests, all the samples were thoroughly cleaned and degreased by sonication in hexane, acetone, and ethanol. Dry heat sterilization at 180 °C for 2 h was performed. The sterilized samples were then placed into cell culture dishes of 60 mm in diameter.

Human osteosarcoma cells U-2 OS (ATCC^®^ HTB-96™) were used for the purpose of fluorescence microscopy. For the observation of cell morphology in the scanning electron microscope, Saos-2 cells (ATCC^®^ HTB-85™) were selected because of their better observability on a rough surface. The U-2 OS cells were resuspended in DMEM (Dulbecco′s Modified Eagle′s Medium, Sigma, D0819) supplemented with 10 vol.% of fetal bovine serum (FBS, Sigma, F7524), Saos-2 cells in McCoy´s 5A medium (Sigma, M8403) supplemented with 15 vol.% of FBS and antibiotics (100 µL/mL). Then, 7 mL of cell suspension were seeded directly onto the sterilized samples, with a seeding density of 12,000 cells cm^−2^. The cells were incubated at 37 °C in atmosphere with 5 vol.% CO_2_.

The first set of samples destined for fluorescence microscopy was fixed after 24 and 96 h of incubation with 4% formaldehyde (20 min at RT). The cell membranes were permeabilized using Tween 20 (0.1%, 15 min), the nuclei were stained by DAPI (0.5 µg mL^−1^, 5 min) and F-actin by phalloidin-TRITC (0.5 µg mL^−1^, 15 min). The fixed samples were observed using an Olympus IX81 inverted fluorescence microscope equipped with a disc scanning unit (DSU). The z-stacks were processed using the maximal intensity projection method. The number of cells after one day was determined by image analysis and compared with the conventionally processed sample and control (cell culture dish).

A commercial LIVE/DEAD^®^ Viability/Cytotoxicity Assay Kit (Thermo Fisher Scientific, Waltham, MA, USA) was applied to the second set of samples for qualitative evaluation. Live and dead cells were distinguished by staining the cells seeded on the samples with 1 μM of calcein AM, 1 μM of ethidium homodimer (EthD-1), and 5 μg mL^−1^ of Hoechst 33342 (Thermo Scientific H1399, Waltham, MA, USA). The cells were incubated at 37 °C in PBS supplied with the dyes for 20 min. Immediately after the staining, fluorescence images were obtained using an Olympus IX81 inverted fluorescence microscope equipped with a confocal spinning unit (CSU).

The third set of samples was fixed after 96 h of incubation with Karnovsky′s fixative (2% formaldehyde, 2.5% glutaraldehyde, and 2.5% sucrose in 0.2M cacodylate buffer) for 1.5 h. After fixation, the samples were rinsed with 0.1 M cacodylate buffer and dehydrated in a series of ethanols (at concentrations of 50, 70, 80, and 100% for 15 min each). At the end, the samples were covered with hexamethyldisilazane (100%, 2 × 10 min), dried at 40 °C for three hours, and sputter coated with 10 nm thick gold layer.

## 5. Conclusions

In this paper, the possibility of employing traditional plasma spray for the preparation of bulk porous titanium intended for orthopedic use was presented. Rods of titanium sprayed on a thin titanium wire exerted a porosity of 15.4 ± 0.8%. Compared to conventionally prepared titanium, a drop in mechanical properties was registered. However, when considering the prepared samples for bone augmentations particularly loaded in compression, plasma spraying provided desirable properties. While a very good strength (UCS of 628 MPa) was preserved, Young′s modulus decreased significantly to 11.6 GPa. Such elasticity perfectly falls into the range of that of human bone. Direct contact in vitro tests indicated the good viability of two types of human bone cells derived from osteosarcoma growing on the plasma-sprayed titanium. To conclude, the prepared samples were revealed to be a possible and easy solution for bone augmentation.

## Figures and Tables

**Table 1:** Mechanical properties (UCS = ultimate compressive strength, BS = bending strength, E = Young′s modulus).

Loading	UCS/BS (MPa)	E (GPa)
compression	628	11.6
bending	161	5.4

**Table 2:** Number of U-2 OS cells adhered on the tested materials after 24 h of cultivation.

Sample	Number of Cells (10^3^/cm^2^)
plasma spraying	9 ±1
ground control	11 ± 3
cell culture dish	15 ± 3

## Supplementary Materials

The following are available online at www.mdpi.com/1996-1944/10/9/987/s1. Figure S1: Powder of sponge titanium destined for plasma spray; Figure S2: Prepared plasma-sprayed titanium: bulk sample and titanium layer on a Ti6Al4V plate.
