# Supplementary material for: Novel Approach in the Use of Plasma Spray: Preparation of Bulk Titanium for Bone Augmentations
Source: Materials (Basel). 2017 Aug 24;10(9):987. doi: 10.3390/ma10090987 (PMC5615642; doi:10.3390/ma10090987)
Supplement: Supplementary file 1 [file materials-10-00987-s001.pdf]

Supplementary Materials: Novel Approach in the Use of Plasma Spray: Preparation of Bulk Titanium for Bone Augmentations

Michaela Fousova, Dalibor Vojtech, Eva Jablonska, Jaroslav Fojt and Jan Lipov

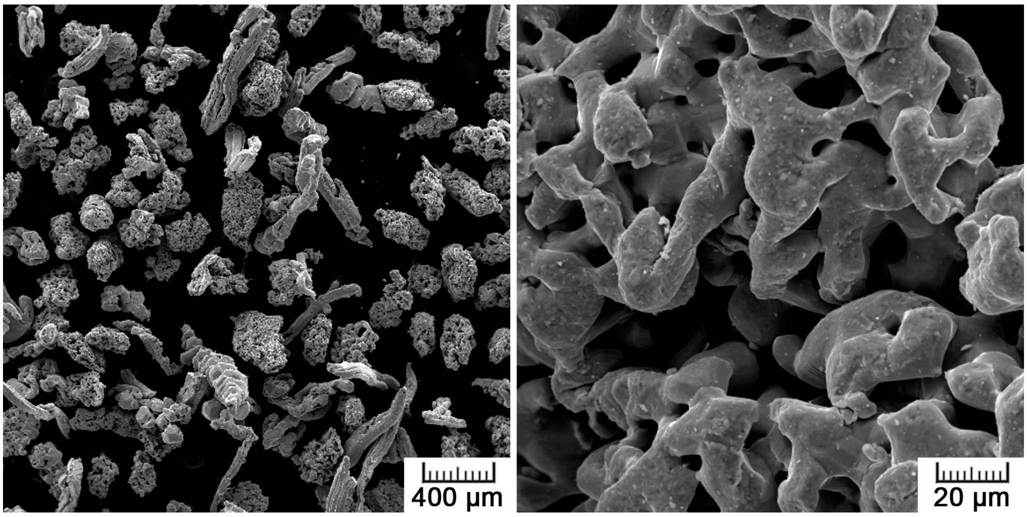

Figure S1. Powder of sponge titanium destined for plasma spray.

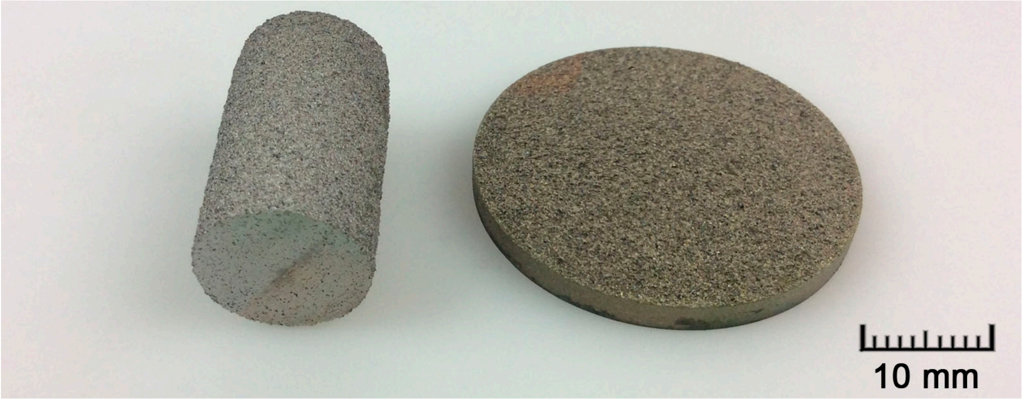

Figure S2. Prepared plasma-sprayed titanium: bulk sample and titanium layer on a Ti6Al4V plate.
